# Heterogeneous melting kinetics in polycrystalline aluminum

**DOI:** 10.1371/journal.pone.0230028

**Published:** 2020-03-10

**Authors:** Yi Liao, Meizhen Xiang, Xiaohua Zhu, Jun Chen, Xia Tian, Liang Ge

**Affiliations:** 1 School of Mechanical Engineering, Southwest Petroleum University, Chengdu, China; 2 Laboratory of Computational Physics, Institute of Applied Physics and Computational Mathematics, Beijing, China; 3 Center for Applied Physics and Technology, Peking University, Beijing, China; 4 College of Mechanics and Materials, HoHai University, Nanjing, China; University of Salento, ITALY

## Abstract

The heterogeneous melting kinetics of polycrystalline aluminum is investigated by a theoretical model which represents the overall melting rate as a functional of the Weibull grain-size-distribution. It is found that the melting process is strongly affected by the mean-grain-diameter, but is insensitive to the shape parameter of the Weibull distribution. The temperature-time-transformation (TTT) diagrams are calculated to probe dependence of the characteristic timescale of melting on the overheating temperature and the mean-grain-diameter. The model predicts that the heterogeneous melting time of polycrystalline aluminum exponentially depends on temperature in high temperature range and the exponent constant is an intrinsic material constant independent of the mean-grain-diameter. Comparisons between TTT diagrams of heterogeneous melting and homogenous melting are also provided.

## Introduction

In single crystals under high heating rate, materials may melt through random nucleation and growth of liquid phase in the crystals (homogeneous melting). In polycrystalline materials, however, melting trends to start from grain boundaries (GBs) and then grow from the outside into the bulk interiors of the grains (heterogeneous melting) [1]. The mechanisms and kinetics of heterogeneous melting have been extensively studied by experiments, theories and molecular dynamics simulations, resulting in a well-established framework for semi-empirical description of the kinetics of the melting process [1–8]. Wherein, Cubeta et al. [8] have used fast scanning calorimetry to investigate low-molecular-weight, bulk-like, polycrystalline films and demonstrate that superheated, non-isothermal, melting kinetics are distinct from those predicted by a direct application of the Wilson-Frenkel theory and its derivatives in common metals. However, most of the previous investigations, have been focused on the motion velocity of an isolated melting front (solid-liquid interface) as a function of temperature. Although the importance is apparent, the overall kinetics of melting process of polycrystalline aggregates, which contains numerous grains and grain boundaries with various sizes and shapes, has not been adequately addressed. In particular, the effects of grain-size-distributions (GSD) on melting kinetics is still not well understood from a quantitative perspective. Our paper mainly focuses on the heterogeneous melting kinetics of the common metal polycrystals within a small time scale by taking polycrystalline aluminum as example. In this work, we propose a theoretical model which represents the overall heterogeneous melting rate as a functional of the Weibull GSD. It allows to quantitatively study the GSD effects on heterogeneous melting of polycrystalline aluminum. The temperature-time-transformation (TTT) diagrams are calculated to probe dependence of the characteristic timescale of melting on the overheating temperature and the GSD.

## Molecular simulations and continuum modeling

**Fig 1:**
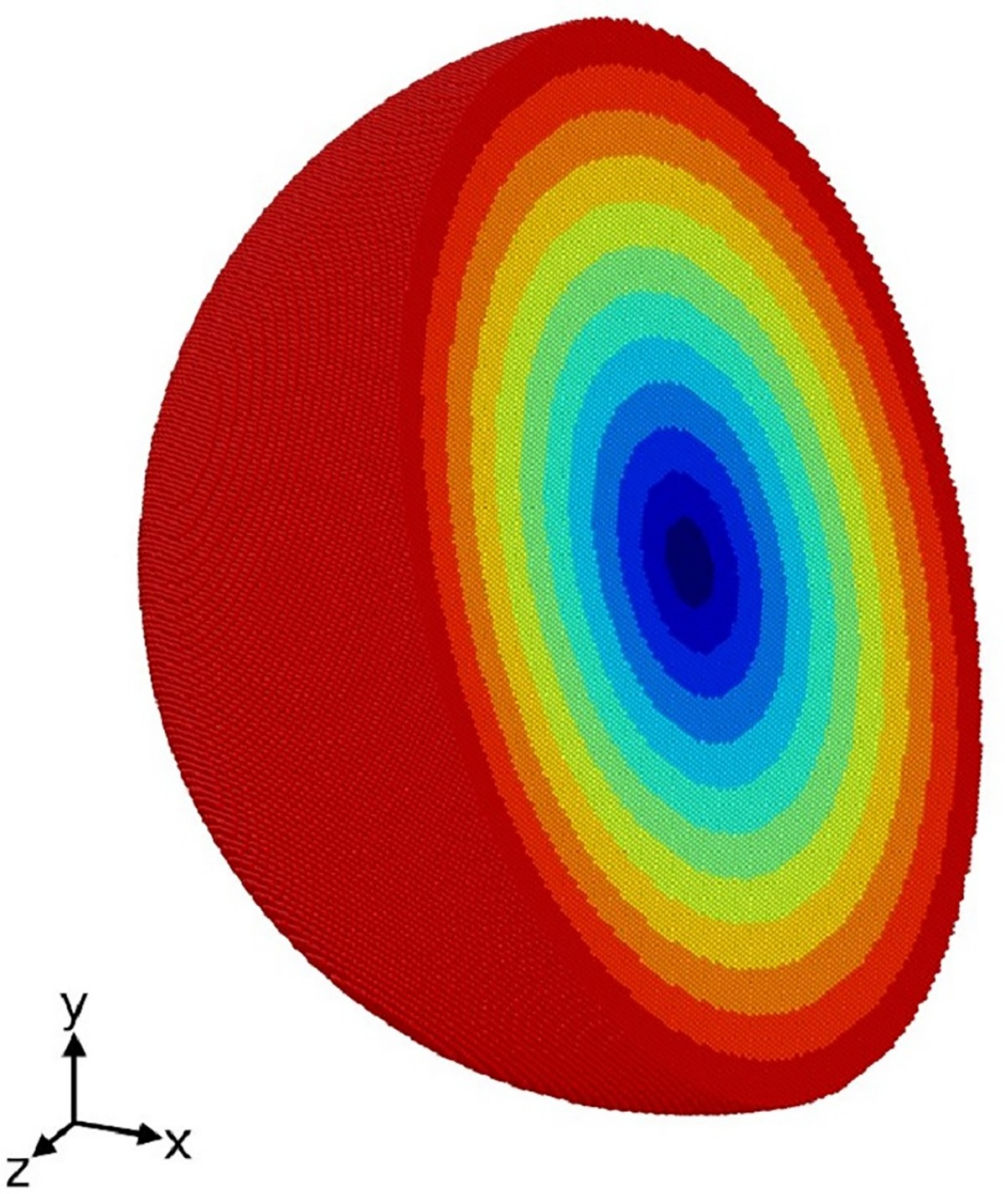
Illustration of the simulation system of a spherical grain. The color indicates different local NPT subsystems. Only half of the sphere is shown. And the initial liquid part is not shown.

Firstly, we focus on the heterogeneous melting of an isolated grain. Generally, polycrystalline materials comprise of grains with various polyhedral shapes. To simplify the modeling, we approximate the polyhedral grains by spheres. Given a polyhedral grain with volume *V*, we approximate the melting behaviors of the grain by that of a spherical grain with diameter *D* = 2×(3*V*/4*π*)^1/3^. We conducted molecular dynamics simulations on melting of an isolated spherical grain. Simulations reported are performed for aluminum (Al), with interatomic interaction described by the embedded-atom method (EAM) in the form suggested in Ref [9]. Some of the properties of the EAM Al material relevant to the melting process are given in Ref [10]. Prior to simulations, the lattice constant for bulk crystal is determined as a function of temperature. The simulation domain is a cubic cell with dimensions 50 nm × 50 nm × 50 nm, filled with a face-centered-cubic (fcc) lattice with the pre-determined lattice constant at the given temperature. The simulation system is divided into two parts: an inner sphere part and the part outside the sphere. We froze the inner sphere and heat the outer part up to 3000 K (far beyond the melting point) in the Berendsen isothermal-isobaric (NPT) ensemble for 30 ps. The basic idea of Berendsen isothermal-isobaric (NPT) ensemble is to assume that a system is coupled with a constant temperature external hot bath. The atomistic system exchanges energy with the hot bath to keep the same temperature as the hot bath. As a result, a solid-liquid coexisting configuration with a spherical interface is created. The radii of the inner sphere is are *R* = 20 nm. The configuration is different from the solid-liquid coexisting configurations in previous works where the interfaces are commonly planar [3–4,7,11]. To investigate the melting kinetics, the solid-liquid coexisting systems are equilibrated at zero pressure and given overheating temperatures (*T* > *T*m). To avoid the local temperature drop at the interface due to the thermal energy transfer to the latent heat of melting, the system is divided into *x* nm-thick shells and the temperature is maintained by the Gaussian thermostat method applied to each individual shell layer, as shown in Fig 1. In the Gaussian thermostat method, a friction force is applied to each atom, and the friction coefficient depends on the difference between the current system temperature and the objective temperature. Similar technique was adopted in Ref [3] for studying plane interface motion.

**Fig 2:**
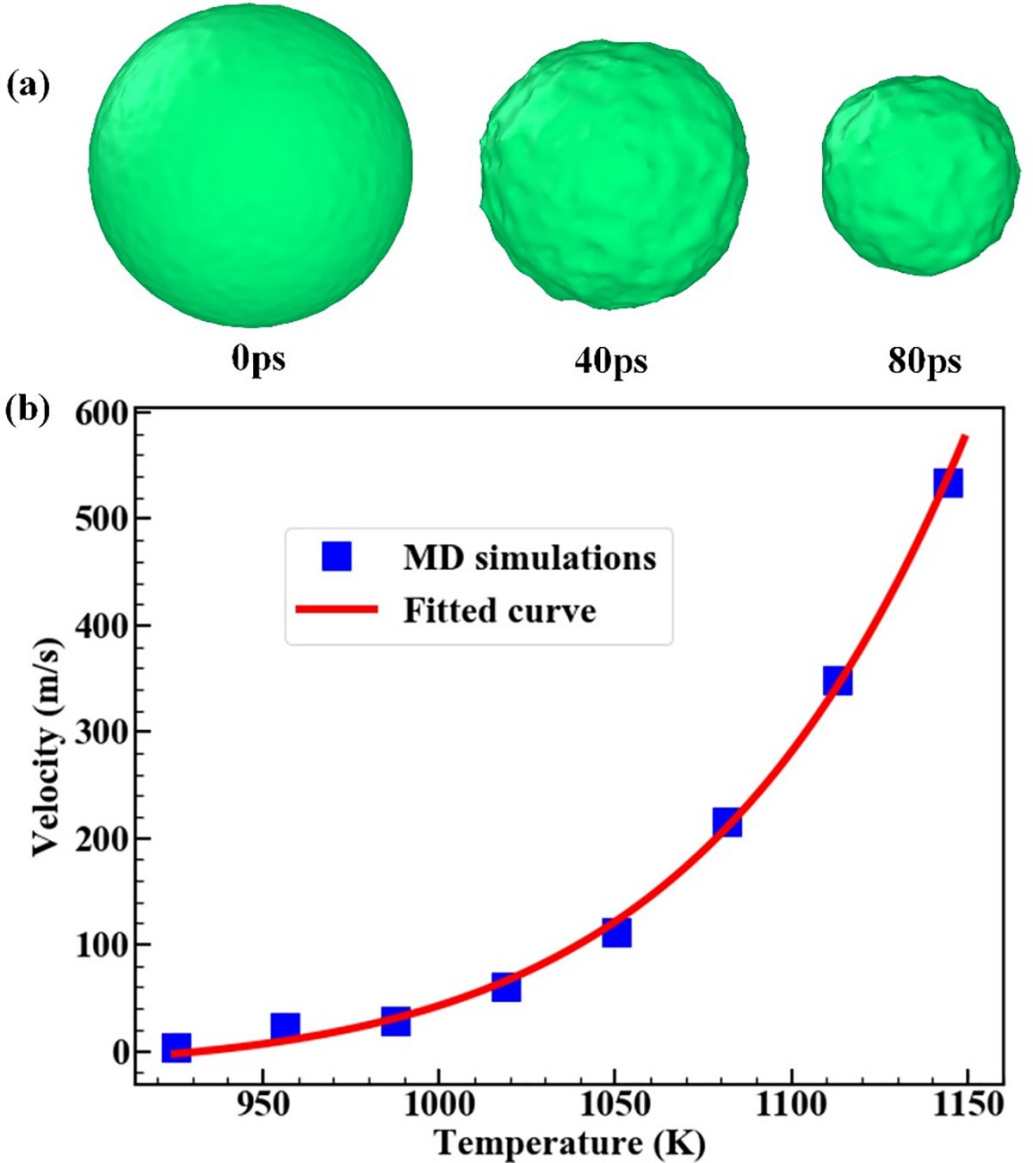
(a) Snapshots taken at different times during the MD simulation; The green surface indicates the solid-liquid interface; The materials outside the surface are in liquid state; The materials inside the surface are in solid state. (b) The motion velocity of the solid-liquid interface as a function of temperature. The squares are direct MD simulation results. The solid line is the fitting curve through Eq (1).

A visual picture of the evolving solid-liquid interface is shown in Fig 2(A) for a MD simulation performed at *T* = 960K. The snapshots show that the melting front shrinks deeper into the crystal (heterogeneous melting) as time goes on. The shape of the moving melting front almost keeps spherical during the melting process. The velocity of the melting front *Ufront* is obtained at different overheating temperature, as shown in Fig 2(B). It is found that the MD simulation data are well fitted by the theoretical model proposed in Ref [4]:

$$U_{front}=a_{U}\sqrt{\frac{3k_{B}T}{M_{atom}}}\left[\exp(b_{U}\frac{\Delta H_{m}}{k_{B}T_{m}}\frac{T-T_{m}}{T})-1\right],\;\forall T\geq T_{m}$$ (1)

Δ*H*m = 10.7 kJ/mol is the melting enthalpy, *T*m = 933 K is the equilibrium melting temperature and *a*_*U*_ = 0.0185, *b*_*U*_ = 13.27 are fitting parameters obtained using the MD simulation data.

According to the MD simulation results, we assume that the melting front is a spherical surface shrinking from the outer GB towards the center. During melting, the grain is divided into two domains: an outer hollow sphere part and an inner sphere part. The outer hollow sphere represents the domain which has been swept by the moving melting front initiated from the GB and is fully melted. The inner sphere is in solid state. Based on this scenario, the heterogenous melting kinetics of an isolated grain with grain diameter *D* is represented by

$$\eta \left(t;D\right)=1-\frac{d{\left(t;D\right)}^{3}}{D^{3}}$$ (2)

where *η*(*t*; *D*) is the overall liquid fraction of the grain at time *t*, *d* is the diameter of the inner sphere which decreases following: $\dot{d}=-2U_{front}$ Under isothermal conditions, *Ufront* keeps constant. Provided initial condition *d*(0; *D*) = *D*, we obtain

$$d\left(t;D\right)=\left\{ \begin{array}{l} D-2U_{front}t,\;\forall t<D/2U_{front}\\ \;0\;,\;\forall t\geq D/2U_{front} \end{array}\right.$$ (3)

Secondly, we model the overall melting kinetics of polycrystalline aggregates with numerous randomly-sized grains on the basis of the model for isolated grain. The overall melting kinetics of polycrystalline aggregates depends on the grain-size-distribution. Here, we use the Weibull distribution to describe the GSD [12,13]. The Weibull probability density function is

$$f\left(D\right)=\frac{k}{\lambda }{\left(\frac{D}{\lambda }\right)}^{k-1}\exp\left[-{\left(D/\lambda \right)}^{k}\right],\;\forall D\geq 0$$ (4)

where *k* is known as the shape parameter and *λ* is the scale parameter which is related to the mean-grain-diameter (MGD) $\bar{D}$ through $\lambda =\bar{D}/\Gamma \left(1+1/k\right)$ where Γ is the gamma function. And the *n*th raw moment of the Weibull distribution is $M_{n}=\int_{0}^{+\infty }f\left(D\right)D^{n}dD=\lambda^{n}\Gamma \left(1+\frac{n}{k}\right)$. Based on the Weibull GSD, the overall transient liquid fraction $\bar{\zeta }\left(t\right)$ is

$$\begin{array}{l} \zeta \left(t\right)=\frac{\int_{0}^{+\infty }f\left(D\right)D^{3}\eta \left(t;D\right)dD}{\int_{0}^{+\infty }f\left(D\right)D^{3}dD}\\ \;=1-\frac{1}{M_{3}}\int_{2U_{front}}^{+\infty }f\left(D\right){\left(D-2U_{front}t\right)}^{3}dD \end{array}$$ (5)

### Applications to polycrystalline aluminum

We apply the model to study melting process of superheated polycrystalline Al (pc-Al). In calculations, the infinite integral in Eq (5) is approximated by $\int_{2U_{front}t}^{+\infty }f\left(D\right){\left(D-2U_{front}t\right)}^{3}dD\approx \int_{2U_{front}t}^{D_{0.99999}}f\left(D\right){\left(D-2U_{front}t\right)}^{3}dD$ where *D*0:99999 is the 0.99999 quantile of the Weibull distribution. The finite integral is then calculated numerically by cumulative trapezoid integral method.

**Fig 3:**
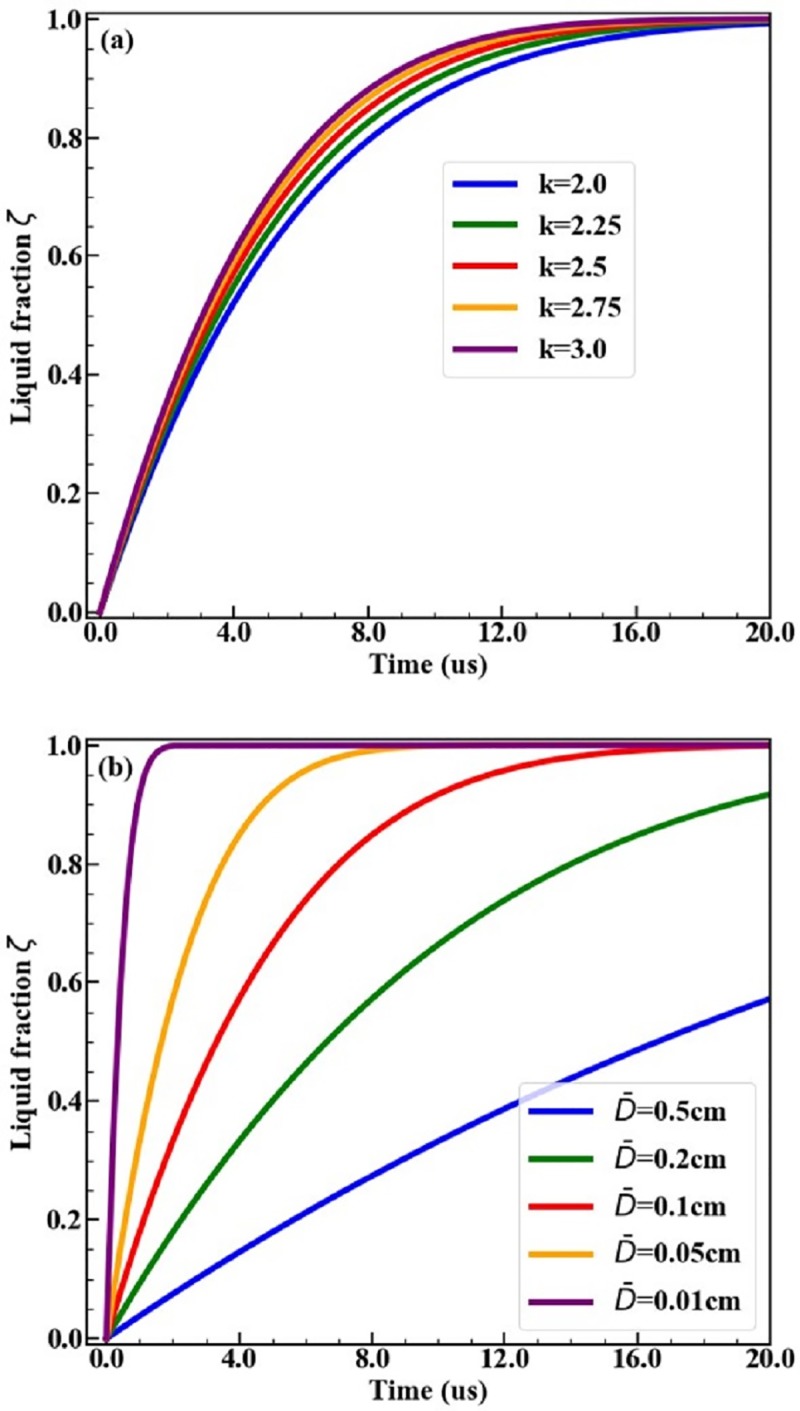
Effects of GSD on melting kinetics under constant temperature *T* = 1128 K. (a) Plots for fixed $\bar{D}=0.1$ cm and various *k*; (b) Plots for fixed *k* = 2.5 and various $\bar{D}$.

Firstly, we probe the effects of the two parameters in the Weibull distribution on the heterogeneous melting process. Fig 3 displays the liquid fraction versus time (*ζ* - *t*) plots under constant temperature *T* = 1128K for pc-Al with different *k* and $\bar{D}$. The shape parameter *k* in the Weibull GSD depends on specific generation methods for polycrystallines. Generally, 2 < *k* < 3 [12]. From Fig 3, for fixed $\bar{D}$, the *ζ* - *t* curves corresponding to different shape parameters *k* almost coincide with each other. However, the curves corresponding to different MGDs deviates apparently. The materials melt faster for smaller MGD. This indicates that the heterogeneous melting process strongly depends on the MGD, but is insensitive to the shape parameter of the Weibull GSD. Therefore, we fix *k* = 2.5 the in following discussions and focus on the effects of MGD.

During heterogeneous melting of an isolated grain, melting rate is proportion to the product of the area of the melting front surface and its motion velocity. The diameter of the spherical melting front steadily decreases in all grains. As a result, heterogeneous melting rate (the slope of the *ζ* - *t* curves) reaches its maximum at the very beginning and then monotonically decreases as melting progresses, as shown in Fig 3. This is quite different from the homogeneous melting process due to random nucleation and growth of liquid phase in bulk crystals. Homogeneous melting process can be divide into “slow-fast-slow” three-stages: in the initial stage, the liquid fraction increases slowly due to lack of growing nuclei; The melting rate (the slope of the curve) increases as the nuclei number increases; In the latter stage, the melting rate slows down again because of lack of mother solid phase [14,15].

**Fig 4:**
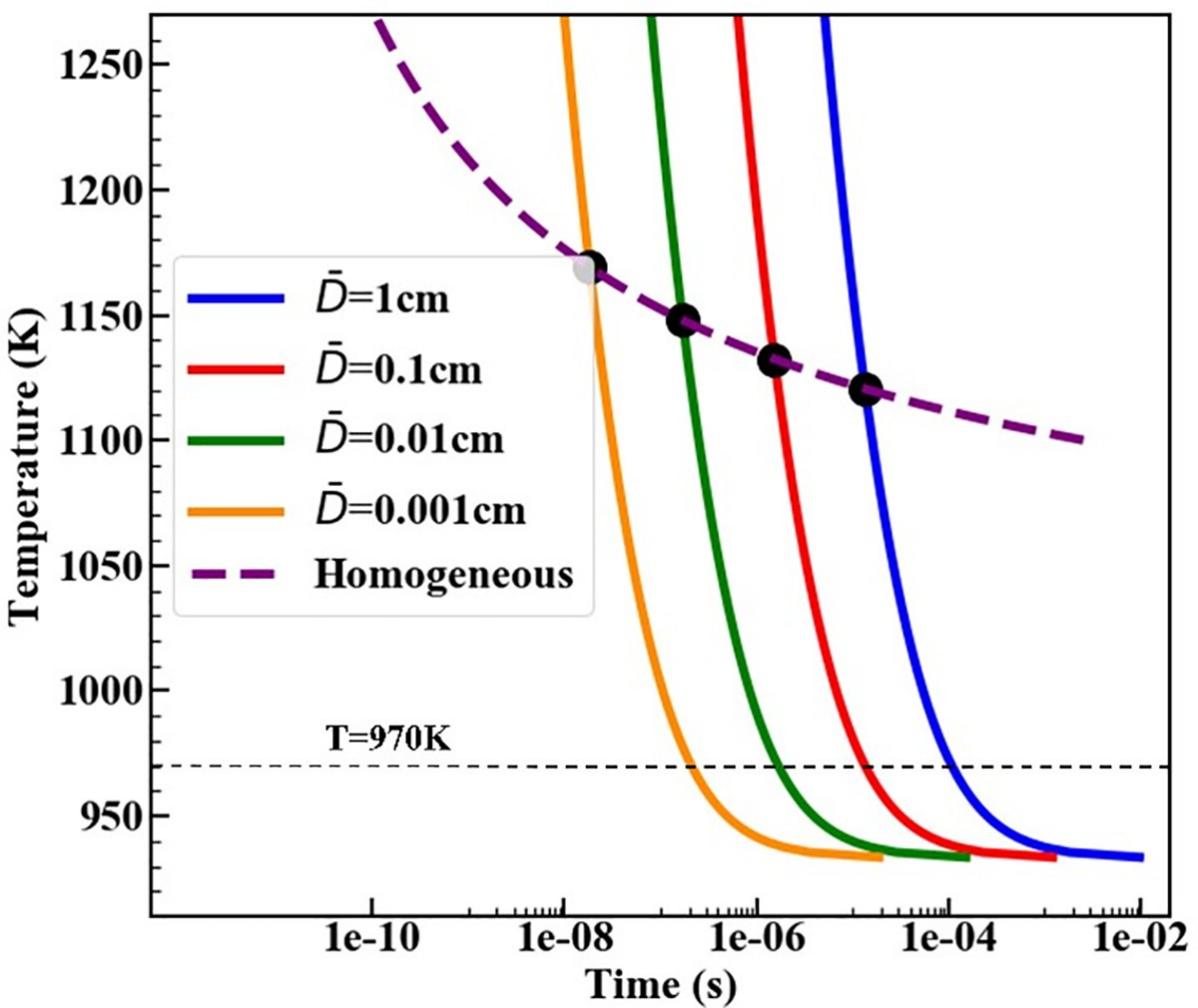
The TTT diagram for polycrystalline Al with various $\bar{D}$.

Temperature-time-transformation (TTT) diagram is an efficient approach to characterize melting kinetics in the whole superheating regime. Provided *ζ* and *T*, Eq 5 is viewed as a nonlinear equation of melting time which is solved by the dichotomy method. Then linking the (*T*, *t*) points corresponding to the same liquid fraction *ζ* = 0.99 forms a curve in the TTT diagram. Fig 4 displays the TTT diagrams of pc-Al with different MGD. It is found that the characteristic timescale of heterogeneous melting rapidly reduces as temperature increases in lower temperature regime. For example, in pc-Al with MGD $\bar{D}=1.0$ cm, the heterogenous melting time reduces from infinity to about 10^−4^ s when the temperature increases from *Tm* = 933K to *T*_*m*_ = 970K. Above 970 K, the melting time lies in the range of 10−6–10^−4^ s. In the high temperature range (*T* > 970K), the TTT curves are approximately straight lines. This indicates that the logarithmic melting time is linearly related to the temperature. In other words, melting time is well described by an exponential function of temperature *t*_*ζ* = 0.99_ = *a*×exp(−*cT*/*T*_*m*_). The TTT curves for different MGDs are parallel to each other for *T* > 970 K, indicating that the exponent rate parameter *c* is an intrinsic parameter which is independent of the MGD. For polycrystalline Al, we calculated that c = 6.7.

Note that, although heterogeneous melting from GBs plays important roles in melting of polycrystallines, homogeneous melting, which is characterized by random nucleation and growth of liquids inside the bulk grain interiors, has been considered as the major melting mechanism near the high superheating under ultrafast heating [1,3]. For comparison purpose, the TTT diagram predicted by the Kolmogorov-Johnson-Mehl-Avrami (KJMA) homogeneous melting model [15,16] is also shown in Fig 4. The parameters for the KJMA model are taken as the same in Ref [15]. It is found that, TTT curves for heterogeneous melting lies below that for homogeneous melting in low temperature regime. In high overheating regime, TTT curves for heterogeneous melting lies below that for homogeneous melting in higher temperature regime. The intersection of the TTT curve for heterogenoeus and homogenoeus melting indicates the critical temperature at which the melting times for the two melting mechanisms are equal. It is shown that the intersection temperature raises as the MGD decreases, which indicates that heterogeneous melting would play the dominant role in a wider superheating temperature range for finer-grained materials. This can be explained by temperature dependence of homogeneous melting and heterogeneous melting. On one hand, increasing temperature would enhance the contributions of homogeneous melting and weaken the contributions of heterogeneous melting. This is because the homogeneous nucleation rate exponentially increases as temperature increases [15], whereas motion velocity of the heterogeneous melting front is much less sensitive to the temperature [3]. On the other hand, the heterogeneous melting rate increases as MGD decreases. As a result, in finer-grained materials, a higher superheating temperature are required to make the overall melting time of homogeneous melting become shorter than that of heterogeneous melting.

## Summary

In summary, we have developed a heterogeneous melting kinetics model for polycrystalline metal within a small time scale. Unlike previous investigations which only consider motion of an isolated melting front, the present work explicitly incorporates Weibull grain-size-distribution and thus can accurately describe the overall heterogeneous melting kinetics of polycrystalline metal aggregates with numerous grain boundaries. By applying the model to polycrystalline Al, we found that the melting process is strongly affected by the mean-grain-diameter, but is insensitive to the shape parameter of the Weibull distribution. We analyzed the TTT diagrams and found that the heterogeneous melting time exponentially depends on temperature in high temperature regime. The exponent constant is an intrinsic material constant which is independent of the mean-grain-diameter. Comparisons between TTT curves of homogeneous melting and heterogeneous melting indicate that heterogeneous melting would play the dominant role in a wider superheating temperature range for finer-grained materials.

## Supporting information

S1 File (ZIP)
